# Medicare Drug Pricing Negotiations: Assessing Constitutional Structural Limits

**DOI:** 10.1017/jme.2024.12

**Published:** 2023

**Authors:** Erica N. White, Mary Saxon, James G. Hodge, Joel Michaels

**Affiliations:** 1. SANDRA DAY O’CONNOR COLLEGE OF LAW, ARIZONA STATE UNIVERSITY, PHOENIX, ARIZONA, USA

**Keywords:** Medicare, Constitution, CMS, Prescription Drugs, Public Health, Agency Deference

## Abstract

A series of structural constitutional arguments lodged in multiple cases against Centers for Medicare and Medicaid Services’ (CMS) authorities to negotiate prescription drug prices via the 2022 Inflation Reduction Act threaten the legitimacy of CMS program and federal agency powers.

Following decades of congressional resistance and inaction, the Inflation Reduction Act (IRA) of 2022^1^ explicitly authorized — for the first time — the Centers for Medicare and Medicaid Services to negotiate prices directly with pharmaceutical companies for drugs provided via Medicare – Part D.^2^ CMS’ resulting Drug Price Negotiation Program (DPNP) seeks to increase consumer access to life-saving prescription medications through lower CMS expenditures and beneficiaries’ out-of-pocket expenses^3^ that spur many Medicare beneficiaries to skip, delay, or ration their prescriptions.^4^ CMS’ announcement in August 2023 of its list of 10 drugs slated for initial price negotiations^5^ are projected to collectively save taxpayers $25 billion by 2031.^6^

Improving access to expensive prescription drugs through price reductions over time promotes communal health. Pharmaceutical manufacturers, however, have challenged the IRA and CMS’ authorities to regulate through manifold lawsuits.^7^ They raise a bevy of “rights-based” constitutional infringements under the First, Fifth, and Eighth Amendments. These specific arguments may seem imposing but are weakly based on federal agency limits to contract with private sector companies. Constitutional rights do not generally restrict agencies like the Department of Veterans Affairs from negotiating prices they pay for private sector services or goods, including drugs. Why should CMS be treated any differently?

Distinct substantive considerations arise from drug manufacturers’ “structural-based” constitutional claims grounded largely in separation of powers principles and framed on a simple premise: that either Congress or CMS exceeded its constitutionally-allocated authorities in crafting the IRA or resulting DPNP provisions. Similar arguments centered on amorphous separation of powers concepts including the non-delegation doctrine, *Chevron* deference, and the major questions doctrine (MQD) have captured the attention of the U.S. Supreme Court and lower courts. Arguments on the scope of the IRA and CMS’ drug pricing provisions promise to do the same. Ultimately, the fate of lower Medicare drug prices over time may rest with the Supreme Court which has shown a penchant for curbing administrative agency authorities notwithstanding clear public health benefits.

## IRA Foundations and Purposes

Medicare as originally conceived in 1965 did not cover self-administered prescription drugs. This coverage gap may seem incredulous, but prescription drugs were a much smaller portion of Medicare recipients’ health care expenses at that time. Escalating drug prices over decades led to calls for significant reforms. In 2003, Congress’ passage of the Medicare Modernization Act created Part D to cover prescription drugs.^8^ Price controls were off the table at that time on the false premise that lower drug prices for consumers would flow from free market principles. U.S. drug prices have increased tenfold between 1980 and 2018, as have CMS’ costs to provide them for Medicare beneficiaries.^9^

Distinct substantive considerations arise from drug manufacturers’ “structural-based” constitutional claims grounded largely in separation of powers principles and framed on a simple premise: either Congress or CMS exceeded its constitutionally-allocated authorities in crafting the IRA or resulting DPNP provisions. Similar arguments centered on amorphous separation of powers concepts including the non-delegation doctrine, Chevron deference, and the major questions doctrine (MQD) have captured the attention of the U.S. Supreme Court and lower courts. Arguments on the scope of the IRA and CMS’ drug pricing provisions promise to do the same. Ultimately, the fate of lower Medicare drug prices over time may rest with the Supreme Court which has shown a penchant for curbing administrative agency authorities notwithstanding clear public health benefits.

Democratic presidential nominee John Kerry campaigned in 2004 to lower drug costs via a Medicare overhaul.^10^ President Barack Obama bandied about the idea in deliberations over the Affordable Care Act (ACA) in 2010. Not until the passage of the IRA in 2022, however, was CMS given unprecedented authority^11^ to identify and select eligible drugs, negotiate with manufacturers to determine fair prices, and implement price reductions over multiple years.^12^ Foregoing formal notice-and-comment rulemaking in favor of agency-issued guidance documents,^13^ CMS selected ten drugs with the highest expenditures for the first round of price negotiations on August 29, 2023.^14^ These initial drugs treat chronic conditions including diabetes, heart disease, and cancers impacting tens of millions of Americans.^15^ The first negotiated prices are slated to take effect in early 2026,^16^ with other drugs following similar processes in ensuing years.^17^

## Emerging Litigation

Following decades of successfully evading price controls, Pharmaceutical Research and Manufacturers of America (PhRMA) did not accept CMS’ DPNP quietly. Yet, drug manufacturers seem to have few options. They must either participate in CMS drug negotiations or face two major consequences: (1) an excise tax up to 95% of a selected drug’s entire U.S. sales; or (2) withdrawal entirely from selling their drugs to CMS for Medicare or Medicaid programs.^18^ Both are considered untenable by manufacturers. High excise taxes would essentially erode all profits from specific drugs. And, with CMS expenditures comprising roughly 40% of the U.S. prescription drug market in 2019,^19^ company walk-aways from CMS’ negotiation table are financially non-viable.^20^ To date, none of the existing drug manufacturers slated for initial price reductions has opted out of negotiations.

What they have done is sued CMS through a bevy of initial cases scattered across U.S. federal courts. Multifarious claims suggest the creation and administration of the DPNP violate core individual rights.^21^ First Amendment claims allege requirements to accept CMS’ rates as “fair” amount to compelled speech. Fifth Amendment arguments hinge on government “takings” of patented intellectual property without just compensation as well as procedural regulatory protections. Excise taxes based on national revenue inspire Eighth Amendment excessive fine arguments. Many legal observers consider these rights-based arguments meritless; to date, no court has ruled favorably on the same.

## Structural Constitutional Arguments

The future of the IRA and CMS’ DPNP may not depend on the Bill of Rights as much as structural constitutional principles reflecting legal strategies endorsed by the pro-business,^22^ regulatory-adverse^23^ U.S. Supreme Court. In recent years, three congruent tenets premised on separation of powers — the nondelegation doctrine, *Chevron* deference,^24^ and MQD — provide a means to dismiss agency interventions that the Court views as overstepping statutory powers or reflecting impermissible congressional delegation of legislative authority. Resulting judicial opinions negate presidential initiatives and threaten agency powers.

Drug manufacturers directly allege that IRA provisions and CMS’ execution run afoul of separation of powers principles.^25^ IRA permissions for CMS to negotiate price reductions are, they argue, assimilate legislating. What is historically known as the nondelegation doctrine holds that Congress cannot broadly delegate constitutional legislative powers to the other two branches. The doctrine had not been relied on since the New Deal era — that is until a 2019 Supreme Court decision, *Gundy v. United States*. In *Gundy*, multiple Justices expressed interest its reviving the doctrine to restrict Congress from allowing agencies to “prescribe rules” related to mandatory registration requirements via the federal Sex Offender Registration and Notification Act.^26^ Since *Gundy*, opponents of administrative agencies have routinely argued nondelegation violations.^27^ Appellate courts have largely rejected these arguments to date provided Congress sets “intelligible principles” for agencies to follow but application of these claims to CMS’ DPNP may soon arrive before a more receptive Supreme Court.

Just as agencies look to legislative history and public health purposes before carrying out their statutes, courts must consider context and designated federal funds in upholding Congress’ responses to health threats facing Americans today. Reviewing statutes outside their broader context, especially related to clear health priorities sustaining the IRA and DPNP, is the true infringement on separation of powers.

Similar constitutional themes emerge under *Chevron* deference and MQD. In two cases, *Loper Bright Enterprises v. Raimondo* ^28^ and *Relentless v. Department of Commerce*,^29^ the Court seeks to clarify or overrule *Chevron* in its 2023-24 term. Under *Chevron* deference, when a federal statute is ambiguous, courts may defer to reasonable agency interpretations of key provisions, thus enabling agencies to fulfill congressional directives.^30^ Given explicit IRA authorizations for CMS to address drug access and health goals, the federal government may invariably raise *Chevron* deference concerning CMS’ choices. The current political environment, however, suggests *Chevron* has fallen out of favor. SCOTUS has not cited *Chevron* to uphold agency decision-making since 2016.^31^ *Chevron’s* demise almost seems inevitable.^32^

Distinct from *Chevron* deference is MQD which challenges diverse agency regulations of “economic and political significance.” Simply put, Congress must speak clearly for federal agencies to achieve “significant” national impacts. Since 2022, the Court has relied on MQD to condemn what it perceives as overbroad agency regulations targeting carbon emissions^33^ and addressing student loan debt burdens.^34^ Similar strategies in prior years denied agencies’ authorities to set COVID-19 economic protections^35^ and implement safety measures.^36^ With Supreme Court backing, lower courts have warmed to evoking MQD to deny agency authority^37^ when asked to expand statutes past largely-accepted underlying justifications^38^ or when companies spend “millions of dollars per year” to comply with regulations.^39^ Under these standards, CMS’ DPNP provisions impacting the massive U.S. pharmaceutical industry seem ripe for MQD applications favoring business, industry, and property rights. Drug manufacturers may allege that Congress must more explicitly authorize a program that significantly limits their revenues and corresponding drug innovations to survive constitutional scrutiny.

## A Cohesive, Structural Approach

SCOTUS’ review of CMS’ DPNP may be months away, but extant litigation in multiple federal circuits may result in disparate applications of anti-agency litigation strategies, mirroring trends in gun restrictions,^40^ climate change responses,^41^ and labor reforms.^42^ Divisive appellate decisions may stymie or stall CMS’ DPNP execution. To survive judicial scrutiny, IRA and DPNP must be considered in light of discrete public health threats of high prescription drug costs, elevating CMS’ purposes of improving health outcomes.^43^ To the extent that courts ignore congressional and agency directives, they usurp the roles of policy-makers in violation of separation of powers.

The nondelegation doctrine, *Chevron* deference, and MQD are triumvirate parts of a litigation strategy to avoid government regulation entirely. Some federal courts are highly skeptical. The nondelegation doctrine, for example, has been called a “Hail Mary” claim;^44^ most appellate courts generally recognize that even the vaguest statutory directives are sufficient to circumvent it. Still, mixed appellate interpretations of when to defer to agencies under *Chevron* and what to label as a “major question” obfuscate agency authorities, limiting or scaling back specific interventions despite congressional or presidential appeals for agency action.^45^

A more promising constitutional path ahead to sustain the IRA and DPNP arises from a parallel trend in judicial review of administrative agency action tied to improving health outcomes. Public health justifications supporting holistic statutory interpretations based on legislative history and other contexts have dissuaded attempts to overrule provisions broadly and allow agencies to follow congressional directives. Courts have favorably interpreted statutes such as the Federal Coal Mine Health and Safety Act,^46^ the Veterans Health Care Act,^47^ or the Family and Medical Leave Act^48^ which all address specific public health concerns. Many of these cases cite the Supreme Court’s ACA decision in *Sebelius* ^49^ where it declined logical interpretations of specific terms, removed from context, to elevate comprehensive regulatory schemes carefully designed to extensively expand health care access. Other courts tend to uphold agency action following “logical extensions”^50^ of accepted congressional charges including protecting the public’s health.^51^

Even the Supreme Court’s recent opinions severely restricting administrative powers recognize the primacy of established public health charges. SCOTUS’ disparate MQD applications — rejecting broad COVID-19 vaccination mandates for industry^52^ but accepting identical requirements for facilities accepting federal funds under CMS requirements^53^ — underscore how separation of powers arguments fall away when courts accept underlying public health justifications behind statutes and federal funding support. Other recent decisions considering CMS rulemaking declined to overrule *Chevron* or meaningfully strip it of broad discretion.^54^ Just as agencies look to legislative history and public health purposes before carrying out their statutes, courts must consider context and designated federal funds in upholding Congress’ responses to health threats facing Americans today. Reviewing statutes outside their broader context, especially related to clear health priorities sustaining the IRA and DPNP, is the true infringement on separation of powers.
